# COHORT DIFFERENCES IN THE ASSOCIATION BETWEEN CHILDLESSNESS AND SOCIAL EXCLUSION IN OLD AGE

**DOI:** 10.1093/geroni/igad104.0222

**Published:** 2023-12-21

**Authors:** Julia Sauter, Marja Aartsen

**Affiliations:** OsloMet, Oslo, Oslo, Norway; OsloMet Oslo Metropolitan University, Oslo, Akershus, Norway

## Abstract

With the progressive institutionalization of Western societies during the first half of the 20th century, socially standardized life trajectories were established, in which certain transitions were expected to be made by a majority of society. These normative transitions, experienced by most individuals at different ages of life, have a temporality implying potential social sanctions for those who carry them out too early, too late or not at all. In earlier born cohorts compared to later born cohorts, the transition to parenthood was an event that was highly valued both socially and institutionally, but a part of society remained childless. This study investigates whether childless people born between 1920 and 1935 were more socially excluded when aged between 60 and 80 than people from the same cohort who transitioned to parenthood. We further examined whether a later life difference in social exclusion between childless people and people with children became smaller in later born cohorts (1940-1955). Gender stratified analyses (N=6441, 54% women) on data from the European Social Survey suggest that, in later life, childless women in the earlier born cohort had less trust in other people and felt less socially included than women who had at least one child. Childless men in the earlier born cohort feel less socially included in later life than childless men in the later-born cohort. This difference persisted for women over the years but became slightly smaller for men suggesting a diminishing importance of children for social inclusion in later life for men, not women.

